# Tracking the footsteps of Burkholderia mallei: determination of the molecular differences and potential resistance genes

**DOI:** 10.55730/1300-0144.5761

**Published:** 2023-12-21

**Authors:** Dilek DÜLGER, Seda EKİCİ, Mehmet DEMİRCİ, Akın YİĞİN, Orkun BABACAN

**Affiliations:** 1 Department of Medical Microbiology, Faculty of Medicine, Karabük University, Karabük, Turkiye; 2 Republic of Türkiye, the Ministry of Agriculture and Forestry, Veterinary Control Central Research Institute, Ankara, Turkiye; 3 Department of Medical Microbiology, Faculty of Medicine, Kırklareli University, Kırklareli, Turkiye; 4 Department of Genetics, Faculty of Veterinary, Harran University, Şanlıurfa, Turkiye; 5 Department of Veterinary, Kepsut Vocational School, Balıkesir University, Balıkesir, Turkiye

**Keywords:** *B. mallei*, chemical biological radiological nuclear threats, glanders, whole genome sequence

## Abstract

**Background/aim:**

Chemical biological radiological nuclear threats are at an important point in the agenda of world health today, as they can cause mass deaths. *B. mallei* attracts attention as a potential biological warfare agent due to its features such as multidrug resistance, a rapid transmission mechanism via aerosol, the absence of a complete treatment protocol for the infection it causes, and the absence of an approved vaccine for protection against the bacteria. *B. mallei* suspect samples must be studied by experienced personnel in biosafety level III laboratories. *B mallei* is a difficult and troublesome pathogen to diagnose and many unknowns about *B. mallei* today. Therefore, the aim of the study was to determine the molecular differences and potential resistance genes of *B mallei* strains.

**Materials and methods:**

Determination of the molecular differences and potential resistance genes of *B mallei* strains with new bioinformatics approaches by comparatively examining the data of 29 B mallei strains, 10 of which were isolated from Türkiye, on the genome list of the National Biotechnology Information Center (NCBI).

**Results:**

According to the genome annotations of the origins, the origin containing the highest number of CDS which is 5172 was found as the 11th strain obtained in Türkiye in 1949. The origin with the highest number of pseudogenes was determined as 23,344 (China 7) origin. Two hundred and eighty-five pseudogenes found in this strain were obtained from a knee effusion in Myanmar. According to chromosome 2 data, *B. mallei* strain was determined as the most similar strain to ATCC 23344, line 11 with NCTC 10229 strain, and SAVP1 strain was determined as the least similar strain. When the antimicrobial resistance gene markers of the isolates included in the study were examined, *amrA and amrB*, *qacG ade, Burkholderia pseudomallei Omp38* were found to be carrying.

**Conclusion:**

In terms of public health, it was thought that the data obtained as a result of our study about *B mallei*, which is defined as a biological weapon, is very valuable for creating treatment protocols to be applied to possible epidemics in the future. In addition, the available genetic epidemiological data of these strains belonging to a category that is dangerous to work with in a laboratory environment were reviewed.

## 1. Introduction

Chemical biological radiological nuclear threats (CBRN) are at an important point in the agenda of world health today, as they can cause mass deaths [1]. Glanders is one of the oldest known diseases and was described by Aristotle in 384–322 BC [2]. Etiologically, the cause of the disease is a zoonotic bacterium called *Burkholderia mallei (B mallei)*. *B. mallei* attracts attention as a potential biological warfare agent due to its features like multidrug resistance, a rapid transmission mechanism via aerosol, the absence of a complete treatment protocol for the infection it causes, and the absence of an approved vaccine for protection against the bacteria [3]. Because of these factors, *B. mallei* was recorded as one of the first biological weapons used during both the First and the Second World Wars and also during the American Civil War [2–4]. It is included in the Bioterrorism Factors and Diseases List (Category B), the Important Dangerous Factors List of the Biological Weapons Convention, and the European Union Bioterrorism Working Group List by the United States Center for Disease Control and Prevention [5].

*B. mallei* is a gram-negative bacteria and horses are the primary carriers of this bacteria. Horses are largely responsible for transmitting the infection to healthy animals and humans. The incubation period may change from days to weeks, and animals may die within a week after the onset of clinical symptoms [6]. The genome of *B. mallei* American Type Culture Collection (ATCC) 23344 is 5.84 Mbp (mega base pair) long and consists of two circular chromosomes. Chromosome 1 contains 3.51 mbp, and chromosome 2 contains 2.33 mbp [5,6]. The analysis of Whole Genome Sequencing (WGS) data, which has been developed in recent years, allows the comparison of different human or animal origins in terms of virulence, pathogenicity, antimicrobial resistance mechanisms, and phylogeny with a single health approach and offers different data [7]. With the new bioinformatics approaches, the aim of this study was to determine the molecular differences of *B. mallei* strains in silico via examining the data about these strains, and their whole genome has been sequenced on the genome list of the National Center for Biotechnology Information (NCBI).

## 2. Materials and methods

### 2.1. Obtaining WGS data of B mallei origins

Among 93 *B. mallei* strains, FASTA data of 29 different *B. mallei* strains having WGS processing in the whole genome list were downloaded from NCBI. Information about these strains is shown in Table 1.

### 2.2. Performing genome annotations

Genome annotation analyzes of 29 different *B. mallei* strains, which were downloaded from the NCBI database, were performed with GeneMarkS version 6.0 software [8]. As a result of genome annotation analyses, total gene, total coding gene sequence (CDS), coding RNA sequence, 5S rRNA, 16S rRNA, 23S rRNA, tRNA, noncoding RNA (ncRNA), and total pseudogene counts of each strain were obtained.

### 2.3. Performing phylogeny analyses

Evolutionary affinities of the lineages with each other were performed with the CSI phylogeny software (https://www.genomicepidemiology.org/) [9].

### 2.4. Identification of antimicrobial resistance gene markers

The presence of antimicrobial resistance markers in the strains was detected with the CARD (https://card.mcmaster.ca/home) online software [10].

## 3. Results

The comparison data was obtained after the genome annotations of the WGS data of 29 *B. mallei* genomes downloaded from the NCBI genome list are presented in Table 2. According to the genome annotations of the strain, the strain containing the highest number of CDS which is 5172 was found as the 11th strain obtained in Türkiye in 1949. The strain with the highest number of pseudogenes was determined as 23,344 (China 7) strains. Two hundred and eighty-five pseudogenes found in this strain were obtained from a knee effusion in Myanmar.

**Figure 1:**
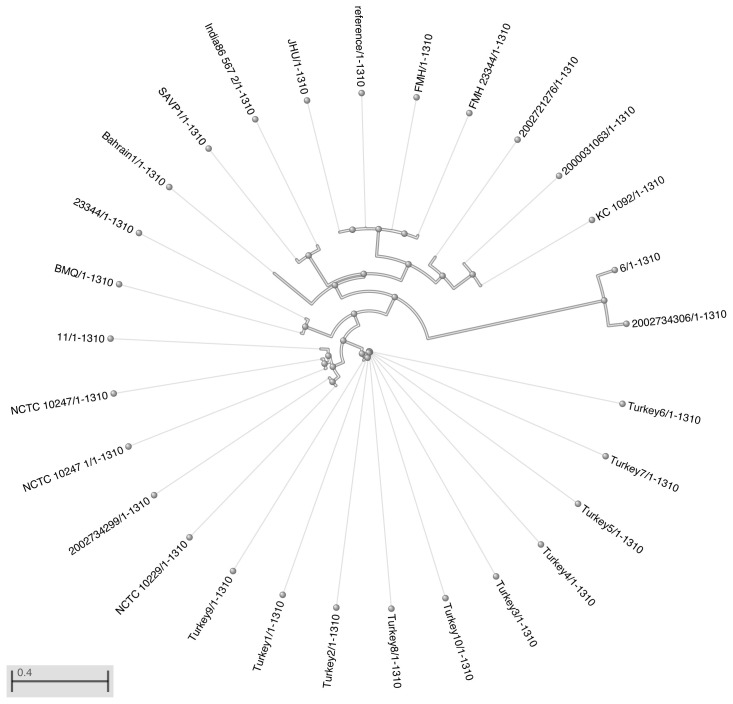
Circular phylogenetic tree of chromosome 1 data from *B. mallei* ATCC 23344 lineage.

**Figure 2:**
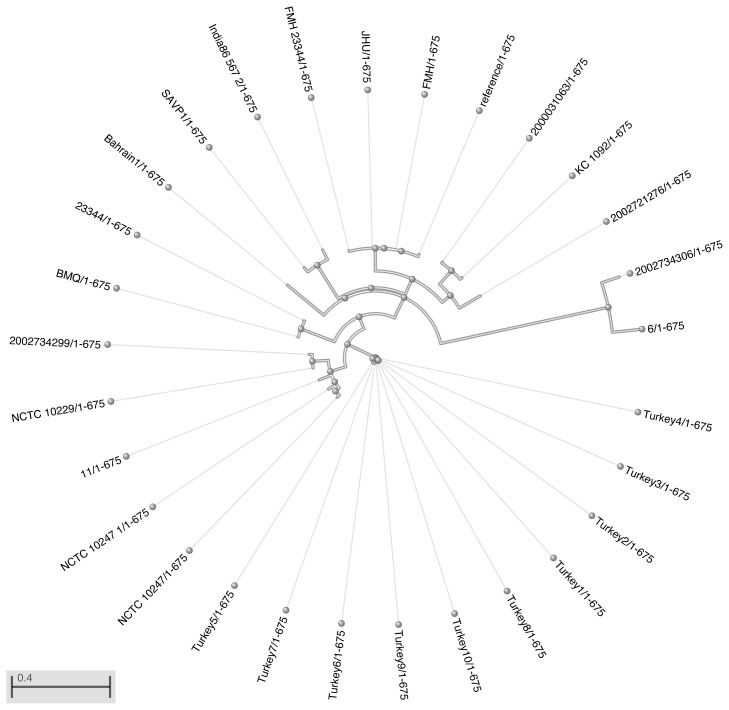
Circular phylogenetic tree of chromosome 2 data from *B. mallei* ATCC 23344 lineage.

As a result of the phylogeny analysis that was performed by checking the single nucleotide polymorphism (SNP) points in the data using whole genome sequencing analysis, similarities of Chromosome 1 and chromosome 2 replicons by the strain of *B. mallei* ATCC 23444 are presented in Table 3. The phylogenetic tree obtained after this analysis is presented in Figure 1 and Figure 2. According to this analysis, human strain 6 was the most similar strain to *B. mallei* ATCC 23344 on chromosome 1 data. The origin with the lowest similarity was found Turkey9 strain. According to chromosome 2 data, the strains most similar to *B. mallei* ATCC 23344 were strain 11 and NCTC 10229. The SAVP1 origin was found to be the one with the lowest similarity. When the antimicrobial resistance gene markers of the isolates which show a perfect and strong match on their chromosomes included in the study are examined, it is seen that the isolates often show gene markers on chromosome 1 that may cause resistance, including aminoglycoside (*amrA and amrB*), disinfectants and antiseptics (*qacG*), and fluoroquinolones and tetracyclines (*adeF*). On chromosome 2, it was found to carry the antimicrobial resistance gene marker “*Burkholderia pseudomallei Omp38*”, which is a general bacterial porin with resistance to fluoroquinolones and tetracyclines (*adeF*) and decreased permeability to beta-lactams. Antimicrobial resistance gene markers of the strains are presented in Table 4.

## 4. Discussion

In addition to its effects on animal and human health such as zoonosis and causing economic losses, the causative bacteria *B. mallei* is also considered as a potential biological weapon agent, increasing the importance of the disease today. The main way to control disease is eradication of animals. Currently, there is no licensed vaccine against B. mallei for humans or animals [11]. *B. mallei* is a gram-negative rod within the Burkholderiaceae family. Although *B. mallei* is easily inactivated by heat and sunlight, its survival time is longer in wet and humid environments. Studies have shown that it has the capacity to survive for approximately 2 weeks outside its host in nature [12]. Spickler [13] reported that *B. mallei* can be destroyed by heating it to 55 °C for 10 min or exposing it to ultraviolet rays. *B. mallei* can survive 3–5 weeks in humid environments, 20–30 days in decaying material, up to 100 days in clean water, and about 6 weeks in contaminated barns [12,14].

Although it is possible for *B. mallei* to be transmitted from horses or other horses to humans during frequent and close contact with infected animals, the number of infected human cases due to *B. mallei* is very low. Although the incidence of transmission from animal to human is low, it should not be ignored that it is an important risk factor for veterinarians and soldiers [15]. Animal-to-animal transmission is possible if the bacteria are taken orally. The glands particularly affect domestic horses and are more resistant than horses, donkeys, and mules. Therefore, a chronic or subclinical form of glanders is observed in horses. Although many horses are chronically infected, they can continue their lives as asymptomatic carriers without showing clinical symptoms [16,17]. Asymptomatic carrier horses are at the forefront of the spread of the disease. Infected animals typically show acute and subacute forms of the disease, respectively. The mode of infection in equidae is uncertain and is generally thought to be caused by contaminated feed or water. Outbreaks are more common in barns where animals are kept together and where managers and drinkers are located. The disease is thought to occur as a result of the growth of the agent in the nasal and trachea region after inhalation or its direct entry into the body via aerosol [13,18]. In addition, direct contact with the secretions and excreta of infected animals, such as skin lesions and abscesses, is another mode of transmission. Therefore, the importance of asymptomatic carriers and thus transmission of infection from them to susceptible animals should not be overlooked [12,19]. Indirect transmission occurs through contact with shared equipment such as blankets, halter, harness, saddle, grooming, and nail clipping material [19]. Contaminated semen is also an effective source of spreading the disease. Although in most cases of gland infection, the infection is confined to only one area, individual cases can sometimes occur in eradicated areas. Glanders, known since ancient times, were eradicated in Australia, Europe, Japan, North America, and some other countries in the early 20th century. The disease has never been reported in New Zealand. Outbreaks or cases still occur sporadically in parts of Asia and the Middle East, North Africa, and Central and South America. The disease is considered endemic in parts of India, Iraq, Mongolia, Pakistan, and Brazil. A significant increase in outbreaks or cases has been reported over the past 25 years, leading to thoughts that there is a reemerging disease [12,17,20]. In many countries, the disease is likely to be underreported or even misdiagnosed. Given the absence of the disease in European countries, it is extremely likely that the disease’s reemergence in the Arab Emirates is due solely to the introduction of infected animals or contaminated semen from endemic areas [21].

Glanders, spread rapidly in Türkiye during the Balkan War, the First World War, and the War of Independence and followed an epidemic course. Between 1925 and 1969, a total of 112 people were reported, 55 of whom were dead. Between 1975 and 1985, infected animals were eliminated by extensive screening to control the disease. The disease was eradicated from Türkiye in 2001 as a result of the “National Glanders Elimination Project” initiated in 2000. According to the latest statements made by the Istanbul Governor’s Office in 2019, 81 horses identified in Türkiye were culled in December 2019. Outbreak in Horses in Büyükada. In 2019, the disease was reported again in horses in Uşak and Bolu, but no human cases were reported [22–24]. In these cases, the routes of transmission or the sources of infection are unknown [24]. B. mallei is an intracellular facultative pathogen that multiplies in phagocytic and epithelial cells and causes infection [25–27]. Memisevic et al. [28] found that the pathogenicity of *B mallei* is due to the PilA and VgrG proteins encoded by the BMA0278 and BMA0446 genes, respectively. In this study, it was observed that PilA and VgrG proteins gave bacteria the ability to attach and support bacterial growth. In an in vitro study using the human respiratory epithelial cell line A549 and mouse, strains with these proteins were eliminated, and it was found that *B. mallei* was easily phagocytosed by the alveoli and reduced pathogenicity [29]. *B. mallei* cannot survive for a long time outside its host, but *B. pseudomallei*, which are closely related to *B. mallei* and are the causative agent of melioidosis in humans, can maintain its viability for a long time in nature. It is estimated that *B. mallei* and *B. pseudomallei* were isolated in an animal host 3.5 million years ago. This is thought to be the result of the evolutions of *B. mallei* [30]. These 2 organisms are known to be resistant to many antimicrobial drugs, including penicillins, polymyxin B, first, second, and third generation cephalosporins [31]. *B. pseudomallei* are inherently resistant to gentamicin, while *B. mallei* is sensitive to gentamicin due to deletion of genes encoding AmrAB-OprA in evolution [31]. *B. mallei* are known to be sensitive to ceftazidime, carbapenems, amoxicillin-clavulanate, piperacillin-tazobactam, doxycycline and trimethoprim sulfamethoxazole (TMP-SMX) [32]. The treatment protocol of *B. mallei* is uncertain due to the culling of sick animals and the rarity of this disease in humans. Multiple antibiotic applications, which are the treatment protocol of *B. pseudomallei*, are applied to humans against *B. mallei* [33]. *B. mallei* are naturally sensitive to gentamicin, but the use of this antibiotic is prohibited as it could potentially be used to treat future infections [32–33]. The results of this study were obtained using a single non-virulent or attenuated strain in vitro.

In our study, the antimicrobial resistance gene markers belonging to 29 *B. mallei* strains with disease-causing characteristics in humans and horses, which were isolated from different regions, whose whole genome sequencing (WGS) process has been completed, were examined. It was determined that they showed resistance to aminoglycosides (*amrA and amrB*), disinfectants and antiseptics (*qacG*), fluoroquinolones and tetracyclines. It has been determined that it carries the antimicrobial resistance gene marker “*Burkholderia pseudomallei Omp38*”, which is a general bacterial porin with reduced permeability (*adeF*) and beta-lactams. Laroucau et al. identified three different lineages (L) for *B. mallei* with the first molecular characterization based on MLVA and SNP analysis. L1 contains only two strains (Türkiye and Arab Emirates). L2 has seven strains, including India, China, Burma, Hungary, Iran, Pakistan, and Türkiye. L3 has 6 strains from Brazil, Hungary, India, Iran, Russia, and America [34, 35]. Falcão et al. [35] reported that the strain of *B. mallei*, which they isolated from horses in the northeast of Brazil, was Türkiye 10 strain. This study shows that interregional migration of *B mallei* strains is possible with globalization and that the origins can acquire different characteristics by evolving.

For genetically homogeneous pathogens such as *B. mallei* species, it is important to examine whole genome sequencing data with new bioinformatics techniques. In this way, a high discrimination power that can show low-level differentiations developing in these strains will be provided, and genomic epidemiological data will be obtained about these strains against epidemics that may develop [36]. It is known that the control of rare random mutations and insertions that occur in the genomes during the proliferation of *B. mallei* with techniques such as WGS is important and can be used for microbial forensic applications [37–39].

## 5. Conclusion

In conclusion, because of globalization, it was considered difficult to geographically restrict diseases as seen in the COVID-19 pandemic. Since B. mallei is a rare agent, it was thought that reporting could not be made in many countries due to the wrong approach and lack of experience, despite the strict restrictions and precautions of the World Organization for Animal Health (WOAH). In addition, it was thought that it should be taken into account that the disease could be seen again due to the civil wars and uncontrolled human and animal movements in Central Asia. Scientists are studying strains of *B. mallei* from different geographic and host origins to gain insight into the evolutionary process of *B. mallei*, a soil bacterium. It was thought that checking the whole genome sequencing data could provide important information about the origin and distribution of these strains. It was thought that the analysis of WGS data with new bioinformatic techniques could provide important data about the virulence, pathogenesis, and resistance mechanisms of these strains and provide precautions against possible epidemics.

## Figures and Tables

**Table 1:** NCBI information of examined *B. mallei* strains in this study.

Origin	NCBI Biosample ID	mega base pair (mbp)	%GC	Chromosome 1 replicon	Chromosome 2 replicon
Turkey2	SAMN03121649	5.59	68.50	NZ_CP009727.1/CP009727.1	NZ_CP009728.1/CP009728.1
Turkey4	SAMN03121651	5.73	68.36	NZ_CP009731.1/CP009731.1	NZ_CP009732.1/CP009732.1
Turkey10	SAMN03121657	5.73	68.36	NZ_CP010348.1/CP010348.1	NZ_CP010349.1/CP010349.1
Turkey7	SAMN03121654	5.73	68.44	NZ_CP009737.1/CP009737.1	NZ_CP009738.1/CP009738.1
Turkey8	SAMN03121655	5.69	68.44	NZ_CP009739.1/CP009739.1	NZ_CP009740.1/CP009740.1
India86-567-2	SAMN03107083	5.69	68.42	NZ_CP009642.1/CP009642.1	NZ_CP009643.1/CP009643.1
11	SAMN03079578	5.91	68.52	NZ_CP009587.1/CP009587.1	NZ_CP009588.1/CP009588.1
Turkey3	SAMN03121650	5.66	68.36	NZ_CP009729.1/CP009729.1	NZ_CP009730.1/CP009730.1
Bahrain1	SAMN05607081	5.78	68.51	NZ_CP017175.1/CP017175.1	NZ_CP017176.1/CP017176.1
2000031063	SAMN02849482	5.87	68.52	NZ_CP008732.1/CP008732.2	NZ_CP008731.1/CP008731.2
2002734306	SAMN03120828	5.41	68.37	NZ_CP009707.1/CP009707.1	NZ_CP009708.1/CP009708.1
2002721276	SAMN03222861	5.78	68.51	NZ_CP010065.1/CP010065.1	NZ_CP010066.1/CP010066.1
BMQ	SAMN02839409	5.63	68.50	NZ_CP008723.1/CP008723.1	NZ_CP008722.1/CP008722.1
23344 (China 7)	SAMN02821273	5.63	68.50	NZ_CP008704.1/CP008704.1	NZ_CP008705.1/CP008705.1
Turkey5	SAMN03121652	5.7	68.41	NZ_CP009733.1/CP009733.1	NZ_CP009734.1/CP009734.1
FMH 23344	SAMN02945023	5.84	68.52	NZ_CP009148.1/CP009148.1	NZ_CP009147.1/CP009147.1
FMH	SAMN03174435	5.84	68.52	NZ_CP009929.1/CP009929.1	NZ_CP009930.1/CP009930.1
JHU	SAMN03174429	5.74	68.47	NZ_CP009931.1/CP009931.1	NZ_CP009932.1/CP009932.1
Turkey6	SAMN03121653	5.75	68.41	NZ_CP009735.1/CP009735.1	NZ_CP009736.1/CP009736.1
6	SAMN02837932	5.65	68.47	NZ_CP008711.1/CP008711.1	NZ_CP008710.1/CP008710.1
Turkey1	SAMN03121648	5.59	68.50	NZ_CP009725.1/CP009725.1	NZ_CP009726.1/CP009726.1
2002734299	SAMN03010440	5.74	68.48	NZ_CP009337.1/CP009337.1	NZ_CP009338.1/CP009338.1
Turkey9	SAMN03121656	5.77	68.44	NZ_CP009741.1/CP009741.1	NZ_CP009742.1/CP009742.1
KC_1092	SAMN03198318	5.66	68.50	NZ_CP009942.1/CP009942.1	NZ_CP009943.1/CP009943.1
ATCC 23344	SAMN02603987	5.84	68.52	NC_006348.1/CP000010.1	NC_006349.2/CP000011.2
NCTC 10229	SAMN02604032	5.74	68.48	NC_008836.1/CP000546.1	NC_008835.1/CP000545.1
NCTC 10247	SAMN02604033	5.85	68.52	NC_009080.1/CP000548.1	NC_009079.1/CP000547.1
NCTC 10247_1	SAMN02798191	5.83	68.52	NZ_CP007802.1/CP007802.1	NZ_CP007801.1/CP007801.1
SAVP1	SAMN02604034	5.23	68.37	NC_008785.1/CP000526.1	NC_008784.1/CP000525.1

**Table 2:** Distribution of data obtained after genome annotations of *B. mallei* chromosome 1.

Origin	Gene (total)	CDS (total)	Gen (RNA)	5S rRNA	16S rRNA	23S rRNA	tRNA	ncRNA	Pseudogene (Total)
Turkey2	4912	4842	70	3	4	3	56	4	211
Turkey4	5091	5021	70	3	4	3	56	4	225
Turkey10	5102	5032	70	3	4	3	56	4	257
Turkey7	5044	4974	70	3	4	3	56	4	222
Turkey8	5010	4939	71	3	4	3	57	4	216
India86-567-2	5079	5011	68	3	4	3	54	4	223
11	5243	5172	71	3	4	3	57	4	257
Turkey3	5025	4955	70	3	4	3	56	4	232
Bahrain1	5121	5051	70	3	4	3	56	4	243
2000031063	5197	5126	71	3	4	3	57	4	256
2002734306	4836	4768	68	3	4	3	54	4	211
2002721276	5116	5045	71	3	4	3	57	4	235
BMQ	5041	4975	66	2	3	2	55	4	282
23344 (China 7)	5015	4949	66	2	3	2	55	4	285
Turkey5	5049	4979	70	3	4	3	56	4	222
FMH 23344	5180	5109	71	3	4	3	57	4	274
FMH	5173	5102	71	3	4	3	57	4	248
JHU	5121	5051	70	3	4	3	56	4	235
Turkey6	5065	4995	70	3	4	3	56	4	227
6	4991	4920	71	3	4	3	57	4	271
Turkey1	4912	4842	70	3	4	3	56	4	218
2002734299	5087	5016	71	3	4	3	57	4	257
Turkey9	5078	5007	71	3	4	3	57	4	225
KC_1092	5019	4953	66	2	3	2	55	4	233
ATCC 23344	5171	5100	71	3	4	3	57	4	248
NCTC 10229	5087	5016	71	3	4	3	57	4	227
NCTC 10247	5178	5108	70	3	4	3	56	4	239
NCTC 10247_1	5161	5091	70	3	4	3	56	4	272
SAVP1	4642	4573	69	2	5	2	56	4	199

**Table 3:** Chromosome 1 and chromosome 2’s phylogeny analysis of *B. mallei* ATCC 23344 origin.

Origin	Chromosome 1 phylogeny		Chromosome 2 phylogeny	
	Current SNP position	Percentage by reference genome	Current SNP position	Percentage by reference genome
Turkey2	3,559,424	101.40	2,146,515	92.31
Turkey4	3,555,225	101.28	2,154,246	92.64
Turkey10	3,539,579	100.84	2,173,899	93.49
Turkey7	3,555,422	101.29	2,171,212	93.37
Turkey8	3,538,401	100.80	2,136,630	91.88
India86-567-2	3,752,764	106.91	2,262,447	97.29
11	3,612,231	102.91	2,387,078	102.65
Turkey3	3,531,176	100.60	2,174,020	93.49
Bahrain1	3,737,232	106.47	2,252,488	96.87
2000031063	3,790,141	107.98	2,570,952	110.56
2002734306	3,547,687	101.07	1,849,753	79.55
2002721276	3,783,874	107.80	2,386,186	102.61
BMQ	3,574,715	101.84	2,229,524	95.88
23344 (China 7)	3,564,461	101.55	2,218,547	95.41
Turkey5	3,539,606	100.84	2,154,817	92.67
FMH 23344	3,580,628	102.01	2,467,562	106.11
FMH	3,591,477	102.32	2,466,008	106.05
JHU	3,611,800	102.90	2,429,114	104.46
Turkey6	3,476,180	99.03	2,181,213	93.80
6	3,498,258	99.66	2,109,987	90.74
Turkey1	3,539,660	100.84	2,146,307	92.30
2002734299	3,567,786	101.64	2,384,063	102.52
Turkey9	2,896,353	82.51	2,117,339	91.05
KC_1092	3,857,770	109.90	2,407,817	103.55
ATCC 23344	3,510,148	100.00	2,325,379	100.00
NCTC 10229	3,591,675	102.32	2,386,903	102.65
NCTC 10247	3,591,548	102.32	2,407,192	103.52
NCTC 10247_1	3,589,569	102.26	2,410,979	103.68
SAVP1	3,657,633	104.20	1,821,075	78.31

**Table 4:** Distribution of antimicrobial resistance gene markers detected in the chromosomes of the examined *B. mallei* strains.

Origin	Chromosome 1	Chromosome 2
**Turkey2**	*amrA, qacG, adeF*	*Omp38, adeF*
**Turkey4**	*amrA, qacG, adeF*	*Omp38, adeF*
**Turkey10**	*amrA, qacG, adeF*	*Omp38, adeF*
**Turkey7**	*amrA, qacG, adeF*	*Omp38, adeF*
**Turkey8**	*amrA, qacG, adeF*	*Omp38, adeF*
**India86-567-2**	*amrA, amrB, qacG, adeF*	*Omp38, adeF*
**11**	*amrA, qacG, adeF*	*Omp38, adeF*
**Turkey3**	*amrA, qacG, adeF*	*Omp38, adeF*
**Bahrain1**	*amrA, amrB, qacG, adeF*	*Omp38, adeF*
**2000031063**	*amrA, amrB, qacG, adeF*	*Omp38, adeF*
**2002734306**	*amrA, amrB, qacG, adeF*	*Omp38, adeF*
**2002721276**	*amrA, amrB, qacG, adeF*	*Omp38, adeF*
**BMQ**	*amrA, qacG, adeF*	*Omp38*
**23344**	*amrA, qacG, adeF*	*Omp38*
**Turkey5**	*qacG, adeF*	*Omp38, adeF*
**FMH 23344**	*qacG, adeF*	*Omp38, adeF*
**FMH**	*qacG, adeF*	*Omp38, adeF*
**JHU**	*qacG, adeF*	*Omp38, adeF*
**Turkey6**	*amrA, qacG, adeF*	*Omp38, adeF*
**6**	*amrA, amrB, qacG, adeF*	*Omp38, adeF*
**Turkey1**	*amrA, qacG, adeF*	*Omp38, adeF*
**2002734299**	*amrA, qacG, adeF*	*Omp38, adeF*
**Turkey9**	*amrA, qacG, adeF*	*Omp38, adeF*
**KC_1092**	*qacG, adeF*	*Omp38, adeF*
**ATCC 23344**	*qacG, adeF*	*Omp38, adeF*
**NCTC 10229**	*amrA, qacG, adeF*	*Omp38, adeF*
**NCTC 10247**	*amrA, qacG, adeF*	*Omp38, adeF*
**NCTC 10247_1**	*amrA, qacG, adeF*	*Omp38, adeF*
**SAVP1**	*amrA, amrB, qacG, adeF*	*Omp38*
